# Respiratory Viral Co-Infections in Pediatric Patients: Clinical Impact and Implications for Healthcare Practice—A Narrative Review

**DOI:** 10.3390/healthcare14091213

**Published:** 2026-04-30

**Authors:** Mariana Șerban (Grădinaru), Gabriela Isabela Verga (Răuță), Silvia Aura Mateescu Costin, Adriana Capăt (Răileanu), Aurel Nechita, Dana Tutunaru, Simona Claudia Cambrea, Mariana Stuparu-Creţu

**Affiliations:** 1 Faculty of Medicine and Pharmacy, Research Center in the Medico-Pharmaceutical Field, “Dunărea de Jos” University of Galati, 800008 Galati, Romania; silviaaura79@gmail.com (S.A.M.C.); aurel.nechita@ugal.ro (A.N.); dana.tutunaru@ugal.ro (D.T.); mariana.stuparu@ugal.ro (M.S.-C.); 2 Emergency Clinical Hospital for Children “Sf Ioan”, 800487 Galati, Romania; 3 St. “Cuvioasa Parascheva” Clinical Hospital of Infectious Diseases, 800179 Galati, Romania; 4 St. “Apostol Andrei” County Emergency Clinical Hospital, 800578 Galati, Romania; 5 Faculty of Medicine & Pharmacy, Ovidius’ University of Constanta, 900527 Constanta, Romania; cambrea.claudia@gmail.com; 6 Clinical Hospital of Infectious Diseases, 900178 Constanta, Romania

**Keywords:** pediatric viral co-infection, respiratory viruses, multiplex PCR, respiratory syncytial virus, human metapneumovirus, viral interference, clinical severity, respiratory viral co-infections

## Abstract

Background: Respiratory viral infections remain a major cause of morbidity and hospitalization in children. The increased use of multiplex molecular assays has improved the detection of simultaneous viral pathogens, raising questions about the clinical relevance of pediatric viral co-infections. Objective: The aim of this study is to synthesize recent evidence on respiratory viral co-infections in children, focusing on clinical severity, healthcare burden, mechanisms, and interpretation of multiplex detections. Methods: This narrative review included studies published between 1 January 2022 and 30 September 2025, identified through PubMed and Web of Science. Eligible studies involved children and adolescents (0–18 years) with laboratory-confirmed respiratory viral co-infections, mainly detected by multiplex PCR. Evidence was synthesized qualitatively across severity, neutral or inconsistent impact, viral interference, and post-acute implications. Results: Forty-five studies were included. The most frequently reported viruses were respiratory syncytial virus (RSV), rhinovirus, adenovirus, influenza virus, and human metapneumovirus (HMPV). Clinical impact was heterogeneous and depended more on specific viral pairings and host factors than on the number of detected viruses. RSV-containing combinations, particularly with HMPV, adenovirus, or rhinovirus, were more often linked to increased respiratory burden, whereas some rhinovirus-associated combinations appeared compatible with viral interference. Conclusions: Pediatric respiratory viral co-infection is clinically relevant but context-dependent. Multiplex results should be interpreted together with clinical, laboratory, and radiological findings.

## 1. Introduction

Respiratory viral infections remain a major cause of morbidity, hospitalization, and healthcare utilization in children worldwide [1,2]. Infants and young children, especially those under five years of age, are particularly vulnerable because of immune immaturity, frequent exposure in community settings, and the high transmissibility of common respiratory viruses [2,3]. Historically, pediatric respiratory disease has often been approached through a “one pathogen, one disease” model, with major attention focused on single agents such as respiratory syncytial virus (RSV), influenza virus, adenovirus, rhinovirus, and parainfluenza virus [3,4]. However, the increasing use of multiplex molecular assays has shown that the simultaneous detection of more than one virus during the same illness episode is common in pediatric practice [5,6].

In the present review, viral co-infection is operationally defined as the simultaneous detection of at least two distinct viral pathogens in the same biological specimen collected during the same clinical episode, preferably by multiplex polymerase chain reaction (PCR) or an equivalent molecular diagnostic assay. This definition was adopted to improve interpretative consistency, although the literature still shows important variation in diagnostic thresholds, sampling methods, and reporting practices [6,7].

The detection of multiple respiratory viruses has important biological and clinical implications. Viral interactions may be synergistic, antagonistic, or neutral, and their net effect depends on both viral and host-related factors [7,8]. Proposed mechanisms include competition for epithelial receptors, modulation of type I and type III interferon pathways, altered cytokine signaling, and disruption of mucosal barrier integrity [8,9,10]. In some cases, these interactions may amplify inflammation and contribute to more severe lower respiratory tract disease; in others, they may reflect viral interference, resulting in reduced replication of one pathogen and a less severe clinical phenotype [8,9].

Despite advances in molecular diagnostics, the clinical relevance of respiratory viral co-infection in children remains uncertain. Some studies have reported longer hospitalization, higher oxygen requirements, and increased intensive care use in co-infected children compared with those with single viral infections [11,12,13]. Other studies have found no major differences after accounting for age, prematurity, comorbidities, or the specific viral pairing involved [14,15]. This inconsistency likely reflects methodological heterogeneity, differences in patient populations, and the lack of a standardized definition of clinically meaningful viral co-infection [7,16].

Particular attention has been directed toward specific viral pairings rather than pooled multiple detections. RSV-containing combinations, especially RSV with human metapneumovirus (HMPV), rhinovirus, or adenovirus, have been more frequently associated with severe respiratory disease in selected cohorts, whereas combinations involving rhinovirus and other respiratory viruses have sometimes been discussed in relation to viral interference rather than severity amplification [8,11,17]. These observations suggest that the effect of co-infection is not uniform and should be interpreted in a pairing-specific context.

The COVID-19 pandemic further complicated this landscape by altering the seasonal circulation of pediatric respiratory viruses. Periods of reduced viral exposure during non-pharmaceutical interventions were followed by atypical waves of viral re-emergence, contributing to the concept of an “immunity gap”, defined as a temporary accumulation of susceptible children with limited recent immune stimulation by common respiratory viruses [18]. However, because the present review focuses on recent literature, these epidemiological changes are considered contextual rather than used as the basis for a formal pre- versus post-pandemic comparison.

Beyond the acute infection, there is a growing interest in whether respiratory viral co-infections influence prolonged viral shedding, immune maturation, later wheezing, or asthma risk, particularly after severe early-life RSV disease [19,20]. At the same time, the widespread use of multiplex PCR panels has created new interpretative challenges in clinical practice, especially in distinguishing true pathogenic co-infection from incidental co-detection or prolonged asymptomatic shedding [7,16].

Against this background, the present narrative review aims to synthesize recent evidence on respiratory viral co-infections in pediatric patients. Specifically, this review seeks to (1) define the current clinical relevance of respiratory viral co-infection in children; (2) analyze whether particular viral pairings are more consistently associated with severe outcomes or greater healthcare resource utilization; (3) summarize mechanistic and immunological insights that may explain heterogeneous clinical expression; and (4) discuss how multiplex viral detections should be interpreted in routine pediatric practice.

## 2. Materials and Methods

### 2.1. Study Design

This manuscript was designed as a narrative review with a structured literature search, rather than as a systematic review. Therefore, a PRISMA flow diagram was not included because the aim was not to perform a formal systematic review or meta-analysis, but to provide a clinically oriented qualitative synthesis of recent evidence.

### 2.2. Eligibility Criteria

Studies were eligible if they included children or adolescents aged 0–18 years with laboratory-confirmed acute respiratory viral infections and reported simultaneous detection of at least two respiratory viruses during the same clinical episode. Particular emphasis was placed on studies using multiplex PCR or equivalent molecular diagnostic assays.

Where available, studies including a comparison group with single viral infection were considered particularly informative. Outcomes of interest included hospitalization, length of stay, oxygen requirement, non-invasive or invasive respiratory support, admission to an intensive care unit (ICU), mortality, and selected immunological or virological parameters such as cytokine profiles, interferon responses, viral load, or cycle threshold values.

Studies were excluded if they were editorials, conference abstracts, animal studies, in vitro studies, or papers lacking extractable data on pediatric respiratory viral co-infection and clinically relevant outcomes. Case reports and small case series were also excluded to reduce publication bias; for the purposes of this review, small case series were defined as studies including fewer than 10 patients.

### 2.3. Information Sources and Search Strategy

Relevant studies were identified through structured searches of PubMed and Web of Science. The search period extended from 1 January 2022 to 30 September 2025. This timeframe was selected to capture recent evidence generated in the contemporary molecular diagnostic era and to reflect the literature most relevant to current pediatric practice.

Search terms included combinations of controlled vocabulary and free-text terms related to viral co-infection, multiple viral detection, respiratory viruses, pediatric patients, disease severity, hospitalization, ICU admission, viral interference, and multiplex PCR. Reference lists of relevant publications were also screened to identify additional eligible studies.

### 2.4. Selection Process

Study selection was performed in a transparent, stepwise manner. First, all records identified through PubMed, Web of Science, and manual reference screening were reviewed by title and abstract to assess their relevance to pediatric respiratory viral co-infections. At this stage, studies were excluded if they clearly did not involve children or adolescents, did not address respiratory viral infections, focused only on bacterial, fungal, or non-respiratory infections, or did not report viral co-detection.

Second, potentially eligible articles underwent full-text assessment. Full texts were evaluated according to predefined inclusion and exclusion criteria, including pediatric population, laboratory-confirmed detection of at least two respiratory viruses during the same clinical episode, and availability of clinically or biologically relevant outcomes. Priority was given to studies using multiplex PCR or equivalent molecular assays and to studies reporting outcomes such as hospitalization, oxygen requirement, respiratory support, ICU admission, mortality, inflammatory biomarkers, cytokine profiles, interferon responses, viral load, or Ct values.

Third, studies were retained for synthesis only when they provided extractable data on respiratory viral co-infection in children and allowed interpretation of clinical severity, healthcare burden, immunological response, or viral interaction patterns. When studies included mixed adult and pediatric populations, only those with clearly identifiable pediatric data were considered. Disagreements regarding eligibility were resolved through discussion and consensus between reviewers.

### 2.5. Data Extraction

Data were extracted using a standardized form developed for this review. For each included study, the following variables were recorded: first author, year of publication, country or region, study design, study population, age group, diagnostic method, definition of co-infection, viral pairing(s), and principal clinical and/or immunological findings.

Particular attention was given to whether studies reported outcomes according to specific viral pairings rather than pooled multiple detections. Clinical variables extracted included length of hospital stay, oxygen requirement, respiratory support, ICU admission, and mortality. Where available, laboratory and immunological variables such as inflammatory biomarkers, cytokines, interferon responses, Ct values, or viral load were also collected.

### 2.6. Data Synthesis

Given the heterogeneity of study design, diagnostic methods, and outcome definitions, evidence was synthesized using a qualitative narrative approach rather than meta-analysis. To improve interpretability and address conflicting findings, the included studies were grouped into four broad analytical domains:

1. respiratory viral pairings associated with increased clinical severity;
2. pairings with neutral, inconsistent, or non-significant clinical impact;
3. findings compatible with viral interference or attenuated severity;
4. evidence extending beyond the acute phase, including prolonged shedding or later respiratory sequelae.

Whenever the available data allowed, interpretation was stratified according to specific viral pairings, patient age, and host-related risk factors, rather than considering all co-detections as a single category.

### 2.7. Methodological Considerations

Because the available literature is dominated by observational studies, the overall evidence base is inherently limited by selection bias, confounding, variable diagnostic sensitivity, and inconsistent adjustment for relevant host factors such as age, prematurity, comorbidities, and nutritional status. Additional heterogeneity arises from differences in sampling strategy, assay composition, positivity thresholds, and the handling of viruses known for prolonged shedding.

These methodological limitations were taken into account throughout the interpretation of the evidence, particularly when assessing whether respiratory viral co-detection reflects true pathogenic co-infection or incidental detection without major clinical significance.

## 3. Results

### 3.1. Studies Selection

This review comprises 45 peer-reviewed studies investigating viral co-infections in pediatric populations. These publications include diverse pediatric age groups, ranging from infants to adolescents and reflect a broad geographical distribution.

The analyzed studies explore a variety of viral combinations, most frequently involving respiratory pathogens such as respiratory syncytial virus (RSV) in association with rhinovirus, influenza virus combined with adenovirus, and metapneumovirus with seasonal coronaviruses. Across these investigations, both clinical severity indicators and immunological response patterns were examined to better understand the implications of multiple viral detection during a single illness episode.

The included literature spans research conducted in the context of widespread implementation of multiplex molecular diagnostic assays, enabling improved detection of concurrent viral pathogens. As a result, the studies collectively provide insight into the clinical presentation, disease progression, and immune response dynamics associated with pediatric viral co-infections in contemporary healthcare settings.

Although study designs, population characteristics, and outcome definitions vary, the available evidence consistently addresses two principal dimensions: (1) the clinical impact of viral co-infections compared with single viral infections, and (2) the underlying pathophysiological mechanisms that may explain differences in disease severity and immune modulation.

This overview establishes the foundation for the subsequent thematic analysis of clinical outcomes, viral–viral interactions, and immunological patterns observed in pediatric patients with multiple viral infections.

### 3.2. Study Characteristics

The reviewed literature comprises 45 studies published between 2022 and 2025, reflecting growing interest in pediatric viral co-infections. Most studies were observational (prospective or retrospective cohorts), with some case–control and multicenter surveillance designs. No randomized controlled trials were identified.

Geographical representation was broad, including Europe, North America, Asia, South America, the Middle East, and Africa. Sample sizes ranged from small single-center cohorts to datasets exceeding 10,000 pediatric cases. Co-infections were most frequently reported in children under five years of age.

Respiratory viruses predominated, particularly RSV, rhinovirus, adenovirus, influenza A/B, and human metapneumovirus, while gastrointestinal viruses such as rotavirus and norovirus were less commonly studied. Frequent viral pairings included RSV–rhinovirus and adenovirus–influenza. Reported prevalence ranged from 15% to 45%, influenced largely by diagnostic methodology, especially the use of multiplex PCR platforms.

Clinical outcomes most often assessed were hospitalization duration, oxygen requirement, ICU admission, and mortality. Several studies also evaluated immunological markers, including cytokine and interferon responses. Definitions of viral co-infection varied across studies, contributing to heterogeneity. Notably, multiple viral detection did not consistently predict increased severity, indicating that clinical impact depends on specific viral combinations and host-related factors.

**Figure 1:**
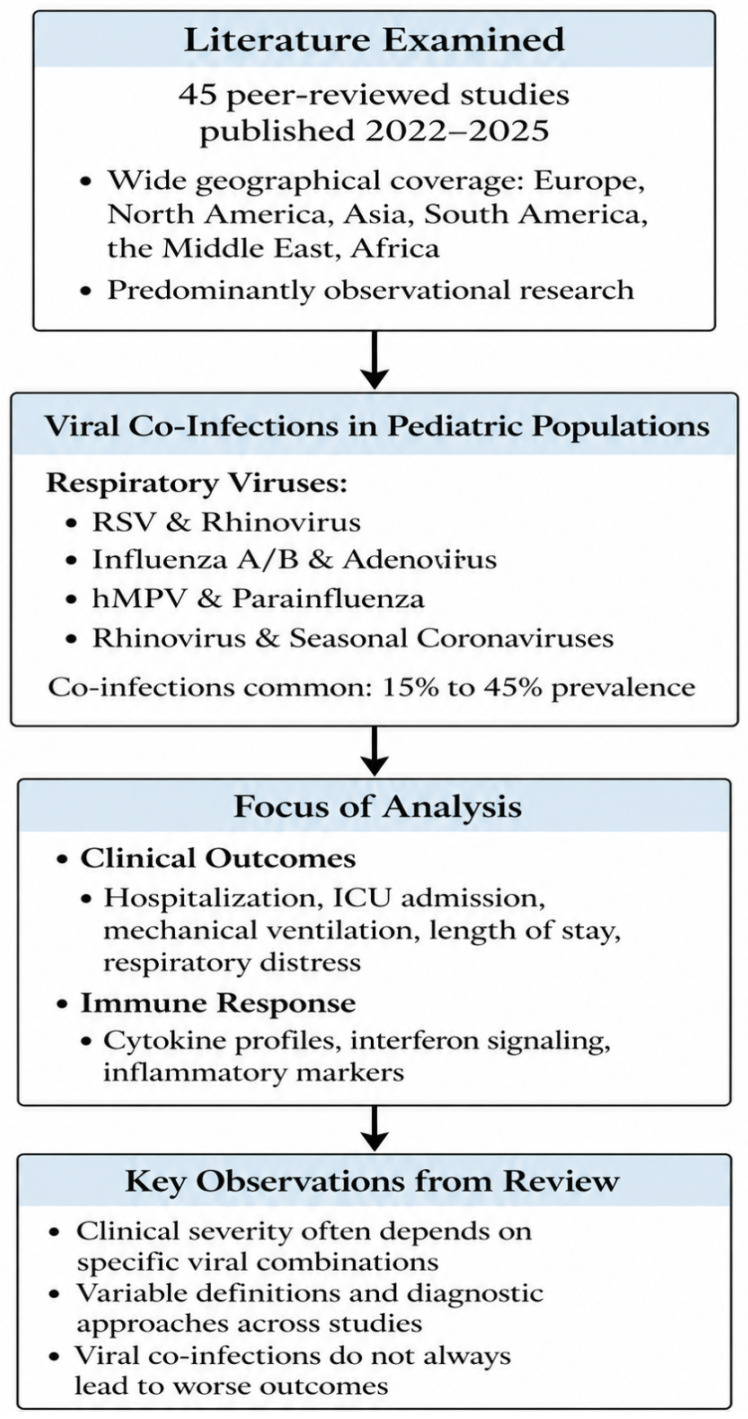
Summary of Reviewed Evidence and Core Domains of Analysis in Pediatric Viral Co-Infections.

Overall, the analyzed studies outline current epidemiological trends and clinical patterns of pediatric viral co-infections while underscoring ongoing methodological variability. To provide a structured overview of the literature examined and the analytical framework applied in this review, the main domains and key observations are summarized in Figure 1.

### 3.3. Type of Viral Interactions and Co-Detection Patterns

Across the 45 studies analyzed, considerable heterogeneity was observed in the type and clinical relevance of pediatric viral co-infections. Most investigations focused on respiratory viruses—particularly RSV, rhinovirus, adenovirus, and influenza—while fewer addressed gastrointestinal or neurotropic combinations such as rotavirus–norovirus or enterovirus–adenovirus.

Viral interactions were variably described as synergistic, antagonistic, or neutral. RSV–rhinovirus and RSV–adenovirus pairings were frequently reported in hospitalized children and were often associated with greater respiratory distress and elevated inflammatory markers. In contrast, rhinovirus–influenza and rhinovirus–coronavirus combinations were sometimes linked to milder disease, consistent with interferon-mediated viral interference.

Recent studies incorporating molecular and immunological profiling have identified distinct cytokine and interferon response patterns associated with specific viral pairings. The widespread use of multiplex PCR and metagenomic sequencing platforms has increased co-detection rates and improved understanding of overlapping viral circulation in both endemic and post-pandemic settings.

A comparative overview of frequently identified viral combinations, study characteristics, and principal observations is presented in Table 1.

Table 1 highlights the marked heterogeneity of viral co-detection patterns in pediatric respiratory disease and the evolving epidemiology observed during and after the COVID-19 pandemic.

Respiratory viruses—including RSV, rhinovirus, influenza, and adenovirus—remain the predominant etiological agents and are frequently co-detected with other viruses or atypical bacteria such as *Mycoplasma pneumoniae*. Virus–bacterium combinations (e.g., RSV with *Haemophilus influenzae* or *Moraxella catarrhalis*) were particularly common in critically ill children, supporting the contribution of mixed infections to severe lower respiratory disease.

Pandemic mitigation measures temporarily suppressed seasonal viruses, followed by increased viral circulation and co-detection once restrictions were lifted, underscoring the dynamic nature of pediatric viral ecology.

Emerging evidence also suggests that environmental exposures and early-life microbiota composition may influence susceptibility and long-term respiratory outcomes. Although respiratory disease predominates, some viruses, including SARS-CoV-2, have been associated with extra-respiratory complications such as neurological involvement.

Overall, these findings emphasize the importance of comprehensive diagnostic approaches—including multiplex PCR and sequencing—to accurately characterize co-infections and inform clinical management.

The available evidence highlights the heterogeneous clinical presentation and biological complexity of multiple viral infections in pediatric patients. Research published between 2022 and 2025 indicates that co-infections—most frequently involving respiratory pathogens such as RSV, rhinovirus, adenovirus, and influenza virus—are associated with a wide spectrum of outcomes, ranging from mild respiratory symptoms to severe disease requiring intensive care, and in some cases, systemic inflammatory responses.

To provide a structured perspective, Table 2 summarizes key clinical and immunological parameters reported across representative studies. These include indicators of disease severity, hospitalization duration, intensive care unit (ICU) admission rates, need for respiratory support, and mortality, together with available data on cytokine expression patterns and interferon responses. The integration of clinical endpoints with immunological findings offers insight into the mechanisms through which viral–viral interactions may modulate disease progression in children.

Co-infections are often linked to increased inflammatory burden, reflected by elevated IL-6, IL-1β, TNF-α, and interferon levels. Severe pairings—such as RSV–parainfluenza or SARS-CoV-2–rhinovirus—have been associated with hyperinflammatory responses and complications including respiratory failure or neurological involvement. In contrast, certain combinations (e.g., RSV–rhinovirus) have been associated with lower cytokine levels and milder outcomes.

Disease severity varies across dual respiratory infections. SARS-CoV-2 combined with RSV or rhinovirus has been associated with higher ICU admission and respiratory support requirements, whereas co-detection of common seasonal viruses (RSV, influenza, HMPV) often results in moderate disease manageable with supportive care.

Immunological responses differ by viral pairing. Interferon-driven profiles may confer partial protection in some combinations, while elevated IL-6, IL-1β, and D-dimer levels correlate with cytokine storm and severe systemic or neurological manifestations.

Although pediatric mortality remains low overall, severe cases highlight the importance of early recognition of hyperinflammatory signatures to guide supportive and immunomodulatory management.

Overall, clinical severity appears to depend more on viral interaction patterns and host immune response than on the mere presence of multiple viruses.

Table 3 summarizes findings from representative studies published between 2022 and 2025 that describe specific viral combinations associated with more severe clinical presentations in pediatric patients. The table integrates reported clinical outcomes with corresponding pathophysiological observations, highlighting viral pairings most frequently linked to increased disease severity.

Across the reviewed literature, certain combinations—particularly those involving RSV in association with other respiratory pathogens—are more commonly associated with intensive care admission, need for respiratory support, or pronounced inflammatory responses. The summarized data illustrate how particular viral interactions may influence disease progression through distinct immunological pathways, including amplified cytokine release or altered interferon signaling.

By organizing these observations comparatively, Table 3 facilitates clearer understanding of which viral pairings appear more consistently related to severe outcomes, while acknowledging the variability inherent in study design, patient characteristics, and diagnostic approaches.

RSV consistently emerges as a central agent, frequently involved in synergistic interactions with influenza, rhinovirus, or adenovirus, and associated with elevated IL-6, IL-8, and TNF-α levels, prolonged oxygen need, and longer hospitalization (Table 3).

Viremia has been identified as a cross-pathogen marker of severity. Systemic viral dissemination in RSV, influenza, or SARS-CoV-2 infections correlates with increased hypoxia, respiratory failure, and ICU admission, suggesting a shared pathway of dysregulated antiviral immunity.

Pandemic-related shifts further illustrate viral interaction dynamics. Suppression of seasonal viruses during mitigation measures coincided with reduced invasive pneumococcal disease, while subsequent relaxation of restrictions led to atypical RSV surges and increased co-circulation—without consistently greater severity across cohorts.

Early life RSV infection, particularly when followed by rhinovirus exposure, has been linked to recurrent wheeze and childhood asthma, supporting the concept of immune imprinting. Although less common, neurotropic co-detections highlight the potential for amplified CNS inflammation in overlapping infections.

Overall, pediatric viral severity reflects a multifactorial model integrating viral combinations, immune maturation, mucosal integrity, and systemic spread, underscoring the importance of preventive strategies such as RSV immunoprophylaxis and influenza vaccination.

Understanding the immunopathological profile of pediatric viral co-infections is essential for clarifying how simultaneous exposure to multiple pathogens modifies host defense. Unlike monoviral infections, which typically follow predictable interferon and cytokine activation pathways, co-infections often generate hybrid or dysregulated immune responses shaped by virus–virus and host–virus interactions.

Recent studies indicate that co-infected children may exhibit distinct cytokine patterns, altered interferon kinetics, and differential innate immune activation. In some cases, these signatures are associated with amplified inflammation and greater clinical severity, including respiratory compromise or multisystem involvement. In others, immune responses reflect viral interference mechanisms that may attenuate replication and moderate disease expression.

These findings suggest that viral co-infections do not represent a uniform entity but rather a spectrum of immune phenotypes influenced by viral pairing, host age, and immune maturity. Table 4 summarizes representative studies (2022–2025), illustrating how specific viral combinations are associated with recognizable immunopathological signatures distinct from single-virus infections.

Table 4 indicates that pediatric viral co-infections are associated with immunopathological signatures distinct from single-virus infections. Several consistent patterns emerge.

Co-infections are commonly linked to amplified innate inflammation, reflected by elevated IL-6, IL-8, TNF-α, CXCL10, and MCP-1, correlating with prolonged oxygen need, severe bronchiolitis, encephalitic presentations, and increased hospitalization. Hyperinflammatory profiles are particularly evident in combinations involving adenovirus, RSV, and SARS-CoV-2.

Interferon responses show context-dependent dysregulation. Excessive activation (e.g., RSV–SARS-CoV-2, enterovirus–HSV) or insufficient signaling—especially in the setting of nutritional deficiencies—may impair viral clearance and worsen outcomes, underscoring the role of host immune status.

Mucosal co-infections (e.g., RSV–HRV, RSV–adenovirus) are often associated with epithelial injury and prolonged shedding, whereas interferon-mediated viral interference in some combinations may attenuate replication and moderate disease severity.

Neurotropic co-detections with elevated cerebrospinal fluid cytokines highlight increased inflammatory risk in CNS involvement.

Overall, these findings support the concept that pediatric viral co-infections produce distinct immune landscapes, with severity shaped by viral pairing and host factors rather than simple additive effects.

This radar chart conceptually illustrates the relative variability and interpretative influence of key domains across studies included in this narrative review, including study design, sample size, diagnostic methods, viral combinations, clinical severity indicators, immunological parameters, geographic diversity, reporting consistency, and follow-up completeness.

Values are presented on a relative 1–5 scale to reflect comparative heterogeneity and interpretative impact. These ratings represent a qualitative appraisal rather than a formal quantitative scoring system.

**Figure 2:**
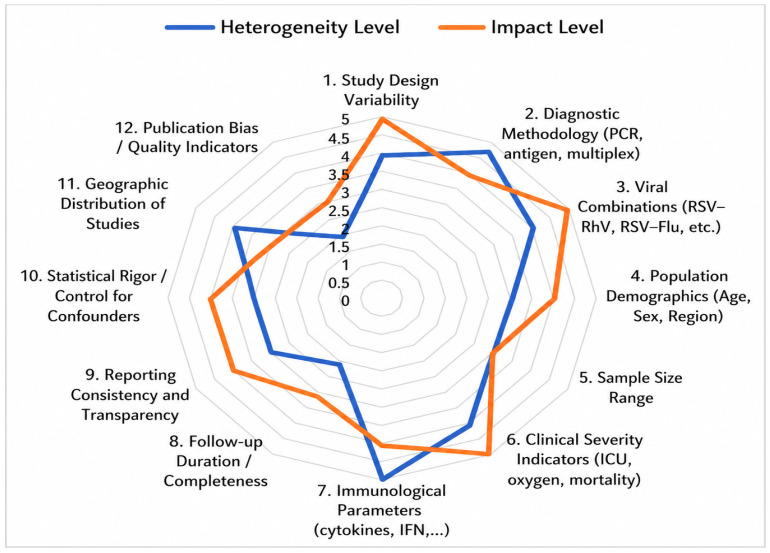
Conceptual Overview of Clinical and Methodological Heterogeneity in Studies on Pediatric Viral Co-Infections (2022–2025).

Figure 2 highlights moderate-to-high variability across clinical and methodological domains in the reviewed literature. The greatest heterogeneity concerns diagnostic approaches and viral combinations, reflecting differences in detection platforms and study comparability. Immunological parameters also vary substantially and influence interpretation of pathophysiological findings.

By contrast, sample size and reporting practices are relatively consistent. Overall, heterogeneity in diagnostic criteria and outcome definitions remains the main challenge in pediatric viral co-infection research, underscoring the need for greater standardization.

## 4. Discussion

### 4.1. Clinical Relevance of Respiratory Viral Co-Infections in Children

This review highlights that respiratory viral co-infection in pediatric patients is a clinically relevant but heterogeneous phenomenon [4,6,14]. The increasing availability of multiplex molecular diagnostics has shown that multiple viral detections are common in children presenting with acute respiratory illness, especially in infants and preschool-aged children [3,4,5,24]. However, the presence of more than one virus does not uniformly predict a worse clinical course [7,8,14]. Rather than acting as a simple additive burden, co-infection appears to modify disease expression in a context-dependent manner influenced by viral pairing, host vulnerability, and the timing of infection [8,14,16,17].

Across the reviewed studies, co-detection rates were highest in young children, likely reflecting both biological susceptibility and high exposure in childcare or family settings [1,2,3,21,24,25,42,43]. Yet the association between co-infection and severity was inconsistent [7,11,14,25,34]. Some cohorts reported higher oxygen requirements, longer hospitalization, and greater ICU use among co-infected patients, whereas others found no major difference after adjustment for confounding variables [1,21,25,34,43]. These discrepancies suggest that pooled analyses of “any co-infection” are of limited clinical value and may obscure meaningful pairing-specific patterns [8,14].

### 4.2. Pairing-Specific Heterogeneity and Severe Outcomes

A major message emerging from the recent literature is that the effect of respiratory viral co-infection depends more on the specific viral pairing than on the mere number of viruses detected [8,11,14]. Among the combinations discussed most often, RSV-containing pairings appear to show the strongest signal for increased clinical burden in selected pediatric cohorts [20,21,33,45]. In particular, RSV associated with HMPV, adenovirus, or, in some settings, rhinovirus has been linked to more severe lower respiratory tract disease, greater oxygen need, longer hospitalization, or more frequent ICU monitoring [20,26,33,40,45].

At the same time, these associations should not be overstated [7,14,20,25,33]. Not every RSV-containing co-infection is severe, and not all studies report the same direction or magnitude of effect [7,14,25]. The variability may reflect differences in age distribution, baseline host vulnerability, viral load, sampling time, and healthcare setting [14,21,25]. Nonetheless, the evidence supports a pairing-specific framework in which some combinations merit closer clinical attention than others [8,14].

The growing emphasis on HMPV is particularly notable [3,20,45]. Although RSV remains the best-established pediatric respiratory pathogen in terms of overall burden, recent evidence suggests that HMPV should be regarded as a pathogen of major concern in co-infection settings rather than as a secondary or incidental agent [20,45]. Severe reported cases and emerging clinical series support its inclusion alongside RSV among the key respiratory viruses relevant to pediatric co-infection surveillance and interpretation [45].

By contrast, some combinations involving rhinovirus have not consistently been associated with greater severity and may in some circumstances reflect viral interference [8,14,17]. This distinction is important, because it argues against treating all multiplex co-detections as biologically equivalent or uniformly prognostic [8,14].

### 4.3. Healthcare Resource Utilization

One of the most clinically important questions raised by respiratory viral co-infection concerns its impact on healthcare resource utilization [1,7,14]. Several observational studies suggest that selected co-infected children, particularly those with RSV-containing combinations or baseline vulnerability, may experience modestly longer hospitalization, greater need for supplemental oxygen, and more frequent escalation to higher levels of respiratory support [1,21,25,34,43]. In specific hospital-based cohorts, co-infection has also been associated with increased ICU use [1,25,43].

However, these trends are not consistent across all studies, and the magnitude of effect varies widely [7,14,24,25,42,43]. This likely reflects differences in study populations, admission thresholds, seasonal epidemiology, and clinical management practices across institutions and countries [14,24,25]. Therefore, co-infection should not be interpreted as an automatic marker of increased healthcare burden [7,14]. Instead, the available evidence supports a more selective approach in which resource implications are considered in relation to specific viral pairings and patient-level risk factors such as infancy, prematurity, chronic cardiopulmonary disease, immunocompromise, or nutritional vulnerability [1,2,16,21,25].

### 4.4. Immunological and Mechanistic Considerations

The biological complexity of respiratory viral co-infection helps explain the observed clinical heterogeneity [8,9,11,14,17]. Viral interactions may be synergistic, antagonistic, or neutral, depending on the order of exposure, viral tropism, replication kinetics, and the maturity of the host immune system [8,14]. Several studies and mechanistic reports point to altered interferon signaling as a central pathway in these interactions [8,16,17].

In some co-infections, impaired or delayed interferon responses may permit enhanced replication of a second virus, contributing to amplified inflammation, epithelial injury, and more severe respiratory compromise [8,11,16,17,20,33]. In others, early induction of antiviral pathways may inhibit replication of the second pathogen, thereby producing a pattern compatible with viral interference [8,14,17]. This mechanism has been discussed particularly in relation to rhinovirus-associated pairings and may help explain why certain combinations are not consistently associated with severe disease [8,14,17].

Cytokine dysregulation also appears relevant [9,10,11,29,41]. Elevated inflammatory markers, including IL-6, IL-8, TNF-α, and related mediators, have been reported in severe pediatric respiratory coinfections, especially in combinations associated with lower respiratory tract disease [11,20,25,29]. However, the available evidence remains incomplete and methodologically variable [7,14,25,34]. Most studies do not provide the temporal or quantitative virological resolution needed to distinguish clearly between causal synergy, sequential infection, and prolonged shedding [4,14].

Host-related factors further shape these immunological responses [2,16,17,26,44]. Age, immune immaturity, prematurity, nutritional status, baseline airway disease, and underlying medical conditions may amplify or attenuate the clinical expression of viral co-infection [2,16,17,26]. For this reason, co-infection should be viewed as a disease modifier operating within a broader host–virus system rather than as an isolated determinant of severity [14,16,17].

### 4.5. Beyond the Acute Illness

At present, the evidence for long-term consequences is more limited than the evidence for acute clinical outcomes, but it is sufficiently suggestive to merit discussion [14,17,37].

Severe early-life RSV disease has been associated with later recurrent wheeze and childhood asthma, and it is plausible that overlapping viral exposures may influence this trajectory through altered immune imprinting or prolonged inflammatory activation [2,17,26,37]. Some studies also raise the possibility that co-infection may affect the duration of viral shedding and the maturation of protective immunity [14,17,26,44]. However, these longitudinal implications remain incompletely defined, and few recent pediatric studies provide robust follow-up data specifically stratified by viral pairing [14,37]. Accordingly, any conclusions beyond the acute phase should be framed as emerging and hypothesis-generating rather than definitive [14,17].

### 4.6. Interpretation of Multiplex Viral Detections in Clinical Practice

A key practical implication of this review is that multiplex viral detection should not be interpreted in isolation [4,7,14]. The identification of two or more respiratory viruses in a child does not automatically indicate clinically meaningful co-infection [4,7,14]. Some viruses, such as adenovirus, bocavirus, and occasionally rhinovirus/enterovirus, may be detected during prolonged shedding or incidental carriage, especially when testing is highly sensitive [4,7,14].

In practice, the likelihood that a multiplex detection reflects true pathogenic co-infection is greater when several elements converge: a compatible acute clinical syndrome, temporal proximity between symptom onset and testing, objective evidence of active disease such as hypoxemia or increased work of breathing, inflammatory biomarker elevation, and radiological findings consistent with active lower respiratory tract involvement [4,7,14,21,25]. When available, lower Ct values, repeated positive testing over a short interval, or detection in lower respiratory specimens may further support clinical relevance [4,14].

Conversely, the additional detection may be incidental when the identified virus is known for prolonged persistence, when the clinical picture is mild or discordant, or when laboratory and radiological findings do not support active lower respiratory infection [4,7,14]. This integrated approach is essential to distinguish true pathogenic co-infection from incidental co-detection and to avoid overinterpretation of molecular results [4,7,14,25].

### 4.7. Clinical Management Implications

From a management perspective, the review supports a cautious but clinically engaged interpretation of respiratory viral co-infection [4,7,14,19]. Multiplex results may contribute to early risk recognition, especially in infants and medically vulnerable children with high-risk viral pairings, but they should not replace clinical judgment [1,4,21,25,43]. Children with RSV-associated co-infections or significant respiratory compromise may require closer monitoring and earlier escalation of supportive care [20,21,33,45].

At the same time, multiplex detection should not automatically trigger antibiotic prescribing unless bacterial superinfection is clinically suspected [4,7,11,19]. Used appropriately, multiplex diagnostics can support antimicrobial stewardship by reducing unnecessary antibacterial treatment in clearly viral syndromes [4,19]. They also have infection-control implications, particularly in hospital settings where co-circulating respiratory pathogens may contribute to transmission risk [1,4,7,24,43].

### 4.8. Limitations of the Evidence Base

The conclusions of this review must be interpreted in light of important limitations in the available literature. First, most studies are observational, retrospective, or cross-sectional, which limits causal inference. Second, definitions of viral co-infection vary substantially across studies, including differences in sampling site, diagnostic assay, positivity threshold, and whether sequential detections are considered part of the same illness episode.

Third, many studies rely on single time-point upper respiratory sampling and do not provide quantitative viral load data, Ct values, or serial testing. This makes it difficult to distinguish true simultaneous pathogenic infection from prolonged shedding. Fourth, outcome reporting remains heterogeneous, with variable definitions of severity, respiratory support, ICU admission, and follow-up duration. Fifth, many studies do not adequately adjust for major confounders such as age, prematurity, chronic disease, nutritional status, and seasonal epidemiology.

An additional limitation of the present review is the selected timeframe. By focusing on studies published between 2022 and 2025, this review prioritizes recent evidence generated using contemporary molecular diagnostics and in the current epidemiological context. However, this approach does not provide a systematic pre-pandemic baseline and therefore limits firm conclusions regarding how the COVID-19 pandemic altered viral circulation patterns in comparison with earlier periods.

### 4.9. Future Directions

Future research should move toward standardized, multicenter pediatric studies using harmonized definitions of respiratory viral co-infection and severity. Priority areas include viral-pair stratification, longitudinal sampling, incorporation of Ct values or quantitative viral load, and integration of immune profiling with clinical outcomes. Such designs would improve the distinction between clinically meaningful co-infection and incidental co-detection.

Longitudinal pediatric cohorts are also needed to clarify whether specific co-infections influence later respiratory morbidity, recurrent wheeze, asthma risk, or immune development. From a clinical perspective, better evidence could support more precise pediatric risk stratification and more rational use of hospital resources during periods of intense viral co-circulation.

## 5. Conclusions

Respiratory viral co-infection in pediatric patients should be regarded as a clinically relevant but heterogeneous phenomenon. Current evidence does not support the assumption that all multiple viral detections are associated with worse outcomes. Instead, the clinical impact depends primarily on the specific viral pairing, host vulnerability, and the broader immunological and epidemiological context.

Among the viral combinations currently discussed in the literature, RSV-containing pairings appear to carry the strongest signal for increased respiratory burden in selected cohorts, with HMPV emerging as an important co-pathogen that deserves greater clinical attention. At the same time, some co-detections, particularly those involving rhinovirus, may reflect viral interference or incidental detection rather than true pathogenic synergy.

The interpretation of multiplex viral detections should therefore be integrated with clinical presentation, laboratory findings, radiological data, and, when available, quantitative or serial virological assessment. Future pediatric studies using standardized definitions and pairing-specific analyses are essential to distinguish clinically meaningful co-infection from incidental co-detection and to improve severity assessment, resource planning, and patient care.

RSV-containing co-infections appear to be associated with greater respiratory burden, particularly in infants and clinically vulnerable pediatric populations. These findings are supported across multiple observational cohorts reporting increased oxygen requirement, longer hospitalization, and more frequent need for intensive care support in selected RSV-associated viral pairings. However, the prognostic value of viral co-detection remains context-dependent and is primarily influenced by the specific viral combination and host-related factors such as age, prematurity, comorbidities, and immune status. Consequently, multiple viral detection alone should not be interpreted as an independent predictor of disease severity without integration of clinical and biological data.

## Figures and Tables

**Table 1:** Type of Viral Interactions and Co-Detection Patterns Among Included Pediatric.

Ref.	Country/Region	Viral Combination(s)	Diagnostic Method	Main Study Focus	Key Observations
[21]	Spain	Broad range of respiratory viruses (RSV and other respiratory pathogens) together with atypical bacteria, mainly *Mycoplasma pneumoniae*, and a smaller proportion of typical bacteria; mixed viral–atypical–bacterial etiologies and involvement of viruses in para-pneumonic pleural effusion.	Extensive microbiological work-up, including respiratory viral panels and tests for typical and atypical bacteria from respiratory and/or blood samples.	Determining the etiology of pediatric CAP in Spain and identifying clinical–radiological predictors of viral vs. atypical vs. typical bacterial pneumonia.	Viruses and *M. pneumoniae* were the main causes of pediatric CAP; viral etiology was associated with younger age, wheezing, lower O_2_ saturation, and absence of radiological consolidation; typical bacteria accounted for a small proportion, supporting more restrictive antibiotic use.
[22]	International	SARS-CoV-2 as the central pathogen; describes virus–host “persistence” interactions rather than virus–virus co-detections.	Review of studies using RT-PCR, serology, cytokine profiling, NF-κB and p38 MAPK analyses.	Proposing a pathophysiological model of long COVID based on SARS-CoV-2 persistence and chronic immune activation.	Suggests that viral or epitope persistence chronically activates NF-κB and p38 MAPK, driving low-grade inflammation and multi-organ symptoms; highlights a possible genetic susceptibility in some pediatric patients.
[23]	International	Focus on RSV and influenza in the context of exposure to ozone, PM_10_, PM_2.5_, PM_0.1_ and indoor pollutants; highlights “virus–pollutant” interaction rather than virus–virus co-infections.	Synthesis of studies combining air pollution monitoring with virological diagnostics for RSV and influenza.	Exploring how air pollutants influence susceptibility to and severity of respiratory viral infections.	Pollutants induce oxidative stress and inflammation, altering immune responses and increasing susceptibility to severe RSV and influenza, particularly in children and other vulnerable groups.
[24]	Germany	Wide viral spectrum: Influenza A/B, RSV, Parainfluenza, HMPV, Adenovirus, seasonal Coronaviruses (OC43, NL63, E229, HKU1), SARS-CoV-2 and Rhino/Enterovirus; marked rise in multiple viral co-infections after 2021, especially in younger children.	Nasal/oropharyngeal samples tested using immunofluorescence (Quidel Sofia) and multiplex PCR respiratory panels (BioFire, Seegene).	Assessing pandemic-related shifts in viral epidemiology, seasonality, and co-infection patterns.	In 2022, viral detections increased four-fold compared to 2019; Rhino/Enterovirus predominated, seasonality was attenuated (especially for Adenovirus and seasonal coronaviruses), and co-infections increased substantially, impacting healthcare burden.
[25]	USA	Respiratory viruses (notably RSV, rhinovirus, bocavirus) together with bacterial pathogens (*H. influenzae*, *M. catarrhalis*, etc.); virus–bacteria co-detections in 52% of LRTI cases; rhinovirus frequently detected as carriage in non-LRTI controls.	Standard testing (bacterial cultures, upper-airway viral PCR) combined with metagenomic next-generation sequencing (mNGS) of tracheal aspirates.	Determining viral and bacterial etiology of severe LRTI using mNGS.	A microbial cause was identified in 92% of children with LRTI; RSV, *H. influenzae* and *M. catarrhalis* contributed disproportionately; virus–bacteria co-detections were very frequent; rhinovirus detection often reflected colonization rather than disease.
[26]	International	RSV-focused; discusses RSV–rhinovirus co-infections and associations with nasopharyngeal bacterial profiles (Hemophilus-, Streptococcus-, Moraxella-dominant) and gut dysbiosis; highlights tripartite virus–virus–microbiota interactions.	Review of studies using 16S rRNA sequencing, metagenomics and microbiome profiling combined with viral diagnostics.	Exploring how early-life microbiota shapes susceptibility and severity of RSV, and later wheezing or asthma.	Specific respiratory/gut microbiota profiles are associated with severe RSV and recurrent wheeze; RSV–rhinovirus co-infections show distinct bacterial signatures, suggesting microbiota modulates infection severity and long-term outcomes.
[27]	Italy	SARS-CoV-2 infection (RT-PCR positive); discusses broader viral encephalitis mechanisms but no viral co-detections in the index case.	SARS-CoV-2 RT-PCR, laboratory tests, brain MRI, EEG and CSF evaluation.	Describing COVID-19-associated encephalitis in a child and mechanisms of neurological involvement.	The child presented with fever and cervical lymphadenopathy, later developing behavioral changes and somnolence; MRI and EEG confirmed encephalitis; rapid improvement with corticosteroids suggests an immune-mediated mechanism triggered by SARS-CoV-2.

CAP = Community-acquired pneumonia; RTI = Respiratory tract infection; LRTI = Lower respiratory tract infection; RSV = Respiratory syncytial virus; HMPV = Human metapneumovirus; PM_10_/PM_2.5_/PM_0.1_ = Particulate matter ≤10 µm/≤2.5 µm/≤0.1 µm; mNGS = Metagenomic next-generation sequencing; PCR = Polymerase chain reaction; CSF = Cerebrospinal fluid; MRI = Magnetic resonance imaging; EEG = Electroencephalography; SARS-CoV-2 = Severe acute respiratory syndrome coronavirus 2; O_2_ = Oxygen.

**Table 2:** Clinical and Immunological Findings Associated with Multiple Viral Infections in Pediatric Patients.

Ref.	Country/Region	Main Viral Combinations	Disease Severity Indicators	Hospitalization/ICU (%)	Respiratory Support (%)	Mortality (%)	Immunological or Biomarker Findings	Key Clinical Observations
[28]	Greece	SARS-CoV-2–RSV, Flu–RhV	Mild–moderate	30% hospitalized	10%	0.2	↑ IL-10, stable IFN-β	Prior exposure to seasonal viruses is associated with less severe COVID-19 co-infections.
[29]	Brazil	RSV–Parainfluenza, RhV–AdV	Severe neurological	50% ICU	40%	2	↑ IL-6, IL-1β, D-dimer	Cytokine storm and encephalitic features triggered by overlapping respiratory viral infections.
[30]	Canada	SARS-CoV-2–RhV	Critical (MIS-C overlap)	62% ICU	55%	3.5	↑ IL-6, IL-10, IFN-γ, ferritin	MIS-C–like hyperinflammation potentiated by dual infection.
[31]	Europe	RSV–RhV	Mild	12% hospitalized	6%	0	↑ IL-10	Nutrient supplementation associated with reduced severity of dual viral infections.

Abbreviations: AdV = adenovirus; Flu = influenza virus; ICU = intensive care unit; IFN-β = interferon beta; IFN-γ = interferon gamma; IL = interleukin; MIS-C = multisystem inflammatory syndrome in children; RhV = rhinovirus; RSV = respiratory syncytial virus; SARS-CoV-2 = severe acute respiratory syndrome coronavirus 2.

**Table 3:** Specific Viral Combinations Consistently Associated with Severe Clinical Outcomes in Pediatric Patients.

Main Viral Combination (s)	Sample/Setting	Indicators of Severity	Outcome Association	Interpretation/Mechanistic Insight	Ref.
RSV + seasonal coronaviruses/influenza (post-lockdown viral co-circulation)	Hospitalized children, Shanghai (COVID-19 period)	Increased RSV detection, atypical seasonality, higher viral load peaks	Moderate LRTI surge without proportional rise in ICU cases	Post-NPI immunity gap enabled viral overlap; co-circulating viruses increased RSV burden through reduced population immunity	[32]
RSV + Influenza/Rhinovirus/Pneumococcus (virus–virus and virus–bacteria synergy)	Mechanistic and clinical review (infants high-risk)	Severe bronchiolitis, airway inflammation, post-RSV wheeze	RSV co-infections linked to more severe acute disease	Impaired IFN responses + epithelial injury enhance susceptibility to co-infecting respiratory viruses and pneumococcus	[33]
RSV + Rhinovirus/Influenza + systemic viremia	Systematic review (pediatric + adult RTIs)	Viremia associated with ↑ respiratory failure, ↑ ICU need	Viremic children had more severe outcomes	Systemic viral spillover reflects immune dysregulation and predicts more severe hypoxia and organ dysfunction	[34]
SARS-CoV-2 + Influenza A/B	COVID–Influenza hospital cohort	Bilateral pneumonia, ↑ oxygen need, complex imaging	More severe respiratory disease than monoinfection	Viral co-infection may intensify lower-airway inflammation and prolong recovery time	[35]
RSV/Influenza + *Streptococcus pneumoniae*	National pediatric surveillance (France)	Decline in RSV and influenza → parallel decline in invasive pneumococcal disease	Virus suppression = major drop in severe bacterial outcomes	RSV/Influenza prime mucosa for pneumococcal invasion; co-infections drive severe pneumonia and sepsis	[36]
Early life RSV + later HRV exposure	Prospective longitudinal pediatric cohort (INSPIRE)	↑ risk of asthma, recurrent wheeze	Severe RSV in infancy → chronic respiratory sequelae	Early RSV “immune imprinting” enhances Th2-skewed responses to later viral exposures	[37]
Enterovirus + HHV-6/HSV (neurotropic co-infections)	CSF meningitis/encephalitis panel (majority pediatric)	Higher severity in HSV/parechovirus cases; rare dual hits	Co-detections linked to increased risk of encephalitis	Syndromic testing reveals mixed CNS infections; combined viral neuroinvasion amplifies inflammatory injury	[38]

Abbreviations: CNS = central nervous system; CoVs = seasonal human coronaviruses; CSF = cerebrospinal fluid; HHV-6 = human herpesvirus 6; HRV = human rhinovirus; HSV = herpes simplex virus; ICU = intensive care unit; IFN = interferon; INSPIRE = Infant Susceptibility to Pulmonary Infections and Asthma Following RSV Exposure; LRTI = lower respiratory tract infection; NPI = non-pharmaceutical intervention; RSV = respiratory syncytial virus; RTI = respiratory tract infection; SARS-CoV-2 = severe acute respiratory syndrome coronavirus 2; Th2 = T-helper type 2 immune response.

**Table 4:** Immunopathological Signatures Associated with Viral Co-Infections in Pediatric Patients.

Main Viral Combination(s)	Distinct Immunopathological Signature Observed	Comparison vs. Single Infection	Clinical Interpretation/Outcome	Ref.
Enterovirus–HSV, EV–HHV-6, occasional dual neurotropic hits	↑ CSF IL-6, ↑ IL-8, ↑ neopterin; microglial activation signature	Greater neuroinflammatory load than single-virus meningitis	Higher risk of seizures, encephalitis, prolonged hospitalization	[39]
AdV + RSV/AdV + HRV (reported in severe AdV pneumonia)	↑ IL-6, ↑ IL-10, ↑ IFN-γ; alveolar macrophage hyperactivation	Co-infections show amplified cytokine storm vs. AdV alone	Severe pneumonia, multi-lobar infiltrates, increased oxygen requirement	[40]
SARS-CoV-2 + other respiratory viruses (MIS-C phenotype)	↑ IL-6, ↑ IL-10, ↑ IFN-γ, ↑ D-dimer; broad hyperinflammatory signature	Markedly stronger hyperinflammation than pediatric COVID-19 alone	MIS-C–like systemic injury; cardiac involvement common	[41]
RSV + Influenza/RSV + seasonal CoVs (post-NPI resurgence)	↑ IL-8, ↑ IL-1β; rapid cytokine bursts during viral overlap	Higher early-phase inflammation vs. isolated RSV in prior years	Increased RTI admissions but no proportional rise in ICU cases	[42]
RSV + HRV/RSV + AdV (Germany, pandemic period)	↑ IL-6, ↑ CXCL10, ↑ neutrophil activation markers	Co-infections exhibited stronger innate activation than mono-infections	Moderate LRTI; increased oxygen need during viral peaks	[43]
RSV + HRV/RSV + seasonal CoVs in post-tonsillectomy children	Altered mucosal immunity: ↓ local IgA, ↑ IL-8, delayed IFN response	Reduced tonsillar barrier lowers antiviral regulation vs. single infections	Higher susceptibility to recurrent viral RTIs, but not increased severity	[44]

Abbreviations: ↑/↓ = increase/decrease compared to monoinfection baseline; AdV = adenovirus; CoVs = seasonal human coronaviruses; CSF = cerebrospinal fluid; CXCL10 = C-X-C motif chemokine ligand 10; EV = enterovirus; HHV-6 = human herpesvirus 6; HRV = human rhinovirus; HSV = herpes simplex virus; ICU = intensive care unit; IFN-γ = interferon gamma; IgA = immunoglobulin A; IL = interleukin; LRTI = lower respiratory tract infection; MIS-C = multisystem inflammatory syndrome in children; NPI = non-pharmaceutical intervention; RSV = respiratory syncytial virus; RTI = respiratory tract infection; SARS-CoV-2 = severe acute respiratory syndrome coronavirus 2.

## Data Availability

No new data were created or analyzed in this study. Data sharing is not applicable to this article.

## Abbreviations

AdV	Adenovirus
ARDS	Acute Respiratory Distress Syndrome
BoV	Bocavirus
CAP	Community-Acquired Pneumonia
CMV	Cytomegalovirus
CNS	Central Nervous System
CoVs	Seasonal Human Coronaviruses (OC43, NL63, 229E, HKU1)
CRP	C-reactive Protein
CSF	Cerebrospinal Fluid
Ct value	Cycle Threshold (viral load indicator)
CXCL10 (IP-10)	C-X-C Motif Chemokine Ligand 10
D-dimer	Fibrin Degradation Product
EEG	Electroencephalogram
ESR	Erythrocyte Sedimentation Rate
EV	Enterovirus
GFAP	Glial Fibrillary Acidic Protein
GI microbiota	Gastrointestinal microbiota
HMPV	Human Metapneumovirus
HRV/RhV	Human Rhinovirus
HSV	Herpes Simplex Virus
HHV-6	Human Herpesvirus 6
IAV/IBV/Flu	Influenza A/Influenza B/Influenza Virus
ICU/PICU	Intensive Care Unit/Pediatric Intensive Care Unit
IFN-α/IFN-β/IFN γ	Interferon alpha/beta/gamma
IFNAR1	Interferon Alpha/Beta Receptor 1
IgA/IgG	Immunoglobulin A/Immunoglobulin G
IL-1β	Interleukin-1 beta
IL-6	Interleukin-6
IL-8	Interleukin-8
IL-10	Interleukin-10
IPD	Invasive Pneumococcal Disease
IVIG	Intravenous Immunoglobulin
LC-PUFA/PUFA	(Long-chain) Polyunsaturated Fatty Acids
LOS	Length of Stay
LRTI	Lower Respiratory Tract Infection
MAPK	Mitogen-Activated Protein Kinase
MCP-1	Monocyte Chemoattractant Protein-1
ME Panel	Meningitis/Encephalitis Multiplex PCR Panel
mAb	Monoclonal Antibody
MIS-C	Multisystem Inflammatory Syndrome in Children
mNGS/NGS	Metagenomic Next-Generation Sequencing/Next-Generation Sequencing
MRI	Magnetic Resonance Imaging
MPXV	Monkeypox Virus
M. catarrhalis	Moraxella catarrhalis
M. pneumoniae	Mycoplasma pneumoniae
NF-κB	Nuclear Factor kappa-B
NK cells	Natural Killer Cells
NP	Nasopharyngeal
NPI	Non-Pharmaceutical Intervention
OAS	2′–5′ Oligoadenylate Synthetase
PCR	Polymerase Chain Reaction
PKR	Protein Kinase R
PIV	Parainfluenza Virus
RSV	Respiratory Syncytial Virus
RT-PCR	Reverse Transcription Polymerase Chain Reaction
RTI	Respiratory Tract Infection
SARS-CoV-2	Severe Acute Respiratory Syndrome Coronavirus 2
S. pneumoniae	Streptococcus pneumoniae
Th1/Th2/Th17	T-helper Cell Subtypes
Tecovirimat	Antiviral for orthopoxviruses
TNF-α	Tumor Necrosis Factor alpha
URTI	Upper Respiratory Tract Infection
Zn	Zinc
